# A Smart Image Encryption Technology via Applying Personal Information and Speaker-Verification System

**DOI:** 10.3390/s23135906

**Published:** 2023-06-26

**Authors:** Shih-Yu Li, Chun-Hung Lee, Lap-Mou Tam

**Affiliations:** 1 Graduate Institute of Manufacturing Technology, National Taipei University of Technology, Taipei 10608, Taiwan; 2 Master Program, Graduate Institute of Mechatronic Engineering, National Taipei University of Technology, Taipei 10608, Taiwan; 3 Institute for the Development and Quality, Macau, Macao 999078, China; 4 Department of Electromechanical Engineering, Faculty of Science and Technology, University of Macau, Macao 999078, China

**Keywords:** novel image encryption, speaker verification, hyper-chaotic system, authorization

## Abstract

In this paper, a framework for authorization and personal image protection that applies user accounts, passwords, and personal I-vectors as the keys for ciphering the image content was developed and connected. There were two main systems in this framework. The first involved a speaker verification system, wherein the user entered their account information and password to log into the system and provided a short voice sample for identification, and then the algorithm transferred the user’s voice (biometric) features, along with their account and password details, to a second image encryption system. For the image encryption process, the account name and password presented by the user were applied to produce the initial conditions for hyper-chaotic systems to generate private keys for image-shuffling and ciphering. In the final stage, the biometric features were also applied to protect the content of the image, so the encryption technology would be more robust. The final results of the encryption system were acceptable, as a lower correlation was obtained in the cipher images. The voice database we applied was the Pitch Tracking Database from the Graz University of Technology (PTDB-TUG), which provided the microphone and laryngoscope signals of 20 native English speakers. For image processing, four standard testing images from the University of Southern California–Signal and Image Processing Institute (USC-SIPI), including Lena, F-16, Mandrill, and Peppers, were presented to further demonstrate the effectiveness and efficiency of the smart image encryption algorithm.

## 1. Introduction

In recent years, people have become accustomed to using online services, such as food delivery and rideshare services (e.g., Uber Eats and Lyft), social media (e.g., Facebook and Twitter), and so on. With convenient and rapid online services, people can exchange their information quickly, but using these services can include many potential risks, such as through the misappropriation of financial transactions and personal information leaks, making the protection of personal information vital and impossible to ignore.

The significance of data protection has escalated across all sectors of society. The recent prevalence of server data breaches has impacted large companies in particular, resulting in the disclosure of users’ personal information. Despite the various measures implemented to process and safeguard data in order to avert attacks and secure digital assets, these measures have often been inadequate to guarantee security, thus posing a critical challenge to privacy and cybersecurity. Data encryption has, therefore, emerged as a critical solution to address this issue. Encryption is a vital technique that transforms readable data into an unreadable format, thereby protecting sensitive information from unauthorized access. The most widely used applications today are centered around sharing and storing images online, making image encryption a crucial tool in the current digital landscape. The intrinsic features of a digital image, including bulk data, high pixel correlation, and redundancy, make image encryption essential for safeguarding sensitive information. Various image encryption schemes have been proposed and studied, including optical transformation [1,2], cellular automata [3,4], DNA coding [5,6], data encryption standards (DESs) [7,8,9], advanced encryption standards (AESs) [10,11], and so on. However, the final three were designed primarily for textual information and may not be suitable for securing digital images.

In 1989, Matthews, a British mathematician, developed the first-ever chaos-based encryption algorithm, which used a logistic map as a key generator [12]. Chaotic behavior can be determined by non-linear dynamic systems, where even a small initial deviation can be exponentially amplified. The intrinsic properties of chaotic systems, such as ergodicity, the sensitive dependence on initial conditions, random-like behaviors, and the mixing effect, have created a natural relationship and structural similarity between chaos and cryptography. This was a significant milestone in the development of chaos-based encryption technology.

Later, in 1998, Fridrich introduced the first general architecture for a chaos-based image cipher [13], which consisted of permutation and diffusion. In the first stage, a two-dimensional area-preserving chaotic map was used to permute the pixels. Then, a discretized chaotic map was employed via the diffusion procedure to modify the pixel values. This architecture has become the most widely adopted structure in chaos-based image encryption algorithms. For example, Chen et al. [14] used a 3D Arnold’s cat map for substitution and Chen’s chaotic system for the diffusion process. In [15], an image encryption algorithm with a permutation–diffusion structure was introduced, and a tent map was used to shuffle the positions of the image pixels. Then, delayed coupled map lattices (DCML) were used to confuse the relationship between the plain and cipher images. In [16], an encryption algorithm based on chaotic technology was introduced that used a logistic map to generate keys. Later, ref. [17] proposed a two-stage encryption structure comprising permutation and diffusion that used chaos-based methods to cipher an image. This architecture has been widely adopted by many image encryption algorithms, based on chaos, and has become the most popular structure. To enhance the two-stage permutation–diffusion architecture, a three-stage architecture was proposed in [18,19,20,21,22,23,24].

Several researchers have proposed modifications to the two-stage encryption architecture, such as the introduction of a one-stage structure [19]. In [19], a one-stage encryption algorithm was suggested that combined both the permutation and diffusion stages. The plain image was divided into multiple blocks that could be permutated individually. Another algorithm was proposed in [20], where the permutation and diffusion stages were processed simultaneously, but the computing unit was the pixels of the image. Furthermore, to prevent attackers from cracking ciphered images using the order of the image pixels, several image encryption algorithms utilizing bit-level permutations were proposed. A novel image encryption algorithm using chaotic maps was introduced in [21], where piecewise linear chaotic maps (PWLCMs) and bit-level permutations were employed. In [22], an image encryption technique based on both Arnold’s cat and logistic maps, as well as using bit-level permutations, was proposed. In [23], a secure chaos-based image encryption algorithm was developed, utilizing a new 2D-LSM with complex chaotic behaviors, and it had good chaotic performance. In [24], a one-stage image protection strategy with a multi-shuffling process was developed, where the permutation stage was strengthened instead of adopting the classical diffusion process. Finally, in [25], an image encryption technique utilizing a 2D logistic-adjusted-sine map was proposed that could perform both confusion and diffusion operations at the bit-level. Furthermore, blockchain techniques have provided decentralized image encryption for smart industries, ensuring cryptographic security, immutability, and data integrity. With its peer-to-peer communication and smart contract functionality, it revolutionized image encryption in the industrial internet of things (IIoT) [26,27,28].

In advanced approaches, image encryption by applying personal information [29,30,31,32], such as user account information and passwords, along with voice, fingerprint, iris, etc., data, has been applied to create biometric keys for protecting personal information. For example, in [29], an encryption technology for personal images was applied, where the user account information and passwords were employed to further generate the required initial condition to drive different chaotic sequences for the keys of permutation as well as for diffusion stages. In [30], an image encryption algorithm was proposed using fractional transforms and scrambling, along with multimodal biometric keys, and it used both iris and fingerprint binary codes for the XOR operation to establish image protection. In [31], voice keys and chaotic maps were used for image encryption, where time-domain and frequency-domain features of the user’s voice were extracted to generate a voice key. In addition, a medical image encryption scheme for secure fingerprint-based authenticated transmission was developed in [32], and a doctor’s fingerprint could be utilized for authenticated transmissions.

**Figure 1:**
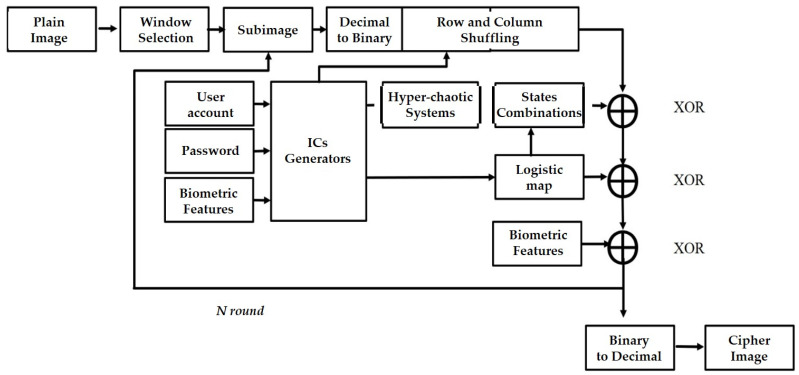
A flowchart of the proposed system.

In this paper, the combination of a speaker verification system and an image encryption system was proposed, where the speaker verification was used as the user authorization system. When users attempted to access the system by inputting their own user account information and passwords, the proposed system asked users to further verify their identification, using I-vectors applied to the image encryption system. This enabled a personal image to be encrypted and protected before the data could be transmitted using their unique keys. The dataset we used was the Pitch Tracking Database from the Graz University of Technology (PTDB-TUG) [33]. The dataset was from the TIMIT corpus (The DARPA TIMIT Acoustic–Phonetic Continuous Speech Corpus), which consisted of 20 English native speakers reading 2342 phonetically rich sentences. A comprehensive flowchart of the proposed system is provided in Figure 1, with the plain image on the left-hand side, and the cipher (encrypted) image on the right-hand side. The proposed plain personal image was divided into several sub-images using a sliding window through appropriate selection. Furthermore, each sub-image was processed using a two-stage encryption procedure with the first process, shuffling, and with the second process, diffusion, where there were three-level encryption processes developed in the diffusion stage. In addition, user account information, passwords, and biometric features (called I-vectors) were employed to generate the corresponding initial conditions for the chaotic system and to generate the necessary chaotic keys for the shuffling and diffusion processes.

This paper is organized as follows: In Section 2, the speaker verification system is described, the proposed image encryption system is introduced in Section 3, and in Section 4, the experimental results and analysis were obtained using the PTDB-TUG database. Finally, the conclusion and related work are discussed in Section 5.

## 2. Speaker Verification System

In this study, there were three stages: the first stage was training, the second stage was enrollment, and the third stage was verification. In the training stage, there were four steps: (2.1) data pre-processing, (2.2) modeling, (2.3) feature-vector extraction, and (2.4) normalization and projection. In (2.1), the feature extraction of the dataset was calculated, and then, in (2.2), the system modeled the features data of 20 people, called the universal background models (UBM). The UBM’s parameters were used to train the total variabilities space (T), and then, in (2.3), T was used to extract the identity vectors (I-vectors). Next, in (2.4), the system normalized and modified the vector space by these two statistical method: within-class covariance normalization (WCCN) and linear discriminant analysis (LDA). The second stage was enrollment, where the user input 15 utterances, and each utterance was 5~7 s, but the user account information and password were also needed. According to user input, the system built a voice model of the user by adjusting the UBM and calculating the I-vector of each user’s voice model. The third stage was verification, during which the test speaker input the user account information and an utterance, and then the system built a test speaker model and compared that to the user’s voice model using cosine similarity: If the similarity between the test speaker model and the user’s voice model passed the threshold, then the system gave the speaker access.

### 2.1. Mel-Frequency Cepstral Coefficients

**Figure 2:**
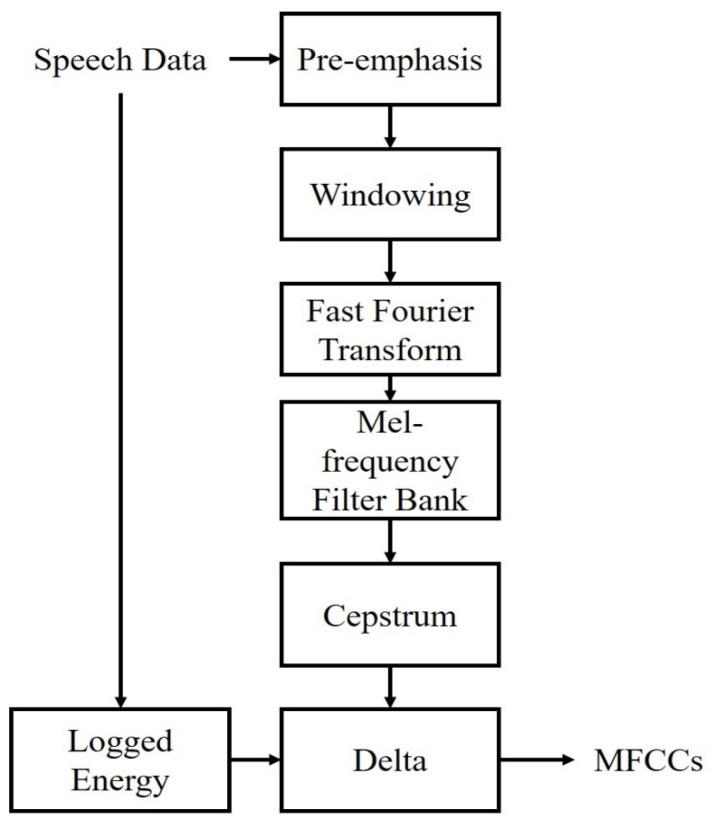
MFCC feature extraction flowchart.

An important technique in voice recognition is the feature-vector extraction. In order to extract the key information in the dataset, the row data must be transformed by signal processing, and to yield a better performance, critical characteristics include being easily measurable, having high robustness to environmental noise, having a minimal effect on the health of the speaker, and being difficult for impostors to mimic. Therefore, the Mel-frequency cepstral coefficients (MFCCs) have been the most general technique used for feature extraction [9]. In our operation, the features extracted using a Hamming window in 25 ms and 20 MFCCs per 10 ms with log energy were calculated [34], and the related MFCC feature extraction flowchart is provided in Figure 2.

### 2.2. Universal Background Models

Identity vectors (I-vectors) were inspired by joint factor analysis (JFA). In the JFA, the supervector (M) represented the speaker utterance and was composed of the speaker, the channel, and the session subspace, and was expressed by $\lambda =\left\{\omega_{i},\mu_{i},\Sigma_{i}\right\}$,i=1,…,M, where $\left\{\omega_{i},\mu_{i},\Sigma_{i}\right\}$ XX means weight, means, and covariance, respectively, and C is the number of Gaussian components.

### 2.3. Identity Vectors

Though the Gaussian mixture model (GMM) has been widely used in the speaker verification field, it requires significant computing resources to calculate statistical parameters that converge. Therefore, the universal background model (UBM) was proposed [35], and the authors used a single and speaker-independent background model to represent a dataset that comprised a large group of people. The whole UBM was composed of two gender-dependent UBMs, and there were 10 males and 10 females. In order to achieve a higher accuracy when modeling the features and better distinguish the differences between speaker models, we used 2048 Gaussian components to train the whole UBM. Gaussian function was defined as follows:

(1) M=m+Vy+Ux+Dz

where *m* is a speaker- and session-independent supervector from the UBM. The speaker subspace was defined by *V* and *D*, where *V* was the eigenvoice matrix and *D* was the diagonal residual. The session subspace was defined by *U*, where *U* was the eigenchannel matrix. The vectors *x*, *y*, and *z* were random variables, and they were speaker- and session-dependent factors in their subspace.

As compared to JFA, the approach of I-vectors in [36] was to replace the speaker- and channel- subspace with only one subspace that contained speaker and channel variabilities simultaneously. The new subspace was referred to as a “total variability space”. It defined a new GMM super vector that depended upon the speaker and vocal track. The total variability matrix contained an eigenvector that had the largest eigenvalue in the total variability covariance matrix (2).

(2) M=m+Tω

where *M* is a parameter that is assumed to be normally distributed with mean vector *m*, ω is a random vector with a standard normal distribution, and *T* is a rectangular matrix of low rank. The ω was the total factor that had a hidden variable, referred to as the I-vector. To find the I-vector, we needed an important statistic: the posterior distribution of the Baum–Welch statistic for the provided utterance, as this defined ω. Because the posterior distribution was a Gaussian distribution mathematically, the I-vector corresponded exactly to the means of the posterior distribution. The statistics of Baum–Welch, extracted using UBM, were also mentioned in [30]. Suppose we had a UBM Ω consisting of a sequence of *L* frames $\left\{y_{1},y_{2},\ldots ,y_{L}\right\}$ and *C* mixed components, and then these components were defined in the feature space of dimension *F*. When we input a provided speech pronunciation using the Baum–Welch statistics, we could acquire the I-vector.

(3) $$N_{c}=\sum_{t=1}^{L}P\left(c|y_{t},\Omega \right)$$

(4) $$F_{c}=\sum_{t=1}^{L}P\left(c|y_{t},\Omega \right)y_{t}$$

First-order Baum–Welch statistics were applied to estimate the I-vector, and we needed these parameters: UBM mean-mixture components, *c* and *P*, where c=1,…,C and $P\left(c|y_{t},\Omega \right)$:

(5) $$F=\sum_{t=1}^{L}P\left(c|y_{t},\Omega \right)(y_{t}-m_{c})$$

where the mean value, *m*, is calculated and *c* is a mixture component of UBM. Then, a provided utterance was input using Equation (6), and the I-vector could be obtained.

(6) $$\omega ={\left(I+T^{t}\Sigma^{-1}N(u)T\right)}^{-1}T^{t}\Sigma^{-1}\tilde{F}(u)$$

where Σ is a diagonal covariance matrix that models the residual variability that was overlooked by the total variability matrix, *T*; N(u) is defined as a diagonal matrix of dimension CF×CF, and F(u) is a supervector of dimension CF×1. These two parameters could be obtained by first-order Baum–Welch statistics, $\tilde{F_{c}}$, with a provided utterance *u*.

### 2.4. WCCN and LDA

After the feature extraction was completed, we analyzed its feature vector and found that because we had used different channels, the feature extraction had unnecessary effects. There were two ways to eliminate these effects and compensate for the signal, within-class covariance normalization (WCCN) or linear discriminate analysis (LDA). LDA analyzes the feature space of the data and defines a new axis to narrow the differences within the classes and maximize the differences between the classes, so that the data is on a well-classified axis and unnecessary directions are eliminated. The purpose of WCCN was to normalize the within-class covariance, so that we could achieve the regional optimal solution more smoothly during the process of convergence.

The WCCN intra-class covariance normalization is a statistical numerical method. The core concept of this method is to linearly separate the imposter and the target speaker, and it has been widely used in SVM modeling. In simple terms, WCCN defines a set of upper limits for classification error metrics and can be expected to minimize the false acceptance and false rejection rates in the training step of an SVM. Once these upper bounds had been minimized, we could find the best solution to this problem, and non-class errors would be minimized accordingly. This allowed us to optimize the hard-margin-separation formalism in SVM. Then, the generalized linear kernel Equation (7) could identify the solution:

(7) $$k(\omega_{1},\omega_{2})=\omega_{1}^{t}R\omega_{2}$$

where *W* is the intra-class covariance matrix calculated by all imposters in the training background, and *R* is a symmetric positive semi-definite matrix. The optimized normalized kernel matrix was $R=W^{-1}$, and we assumed that all speech fragments of a provided speaker belonged to the same category. The calculation of the intra-class covariance matrix used Equation (8) to calculate the following:

(8) $$W=\frac{1}{S}\sum_{s=1}^{S}\frac{1}{n_{s}}\sum_{i=1}^{n_{s}}(\omega_{i}^{s}-\varpi_{s}){\left(\omega_{i}^{s}-\varpi_{s}\right)}^{t}$$

where S is the number of speakers and $n_{s}$ is the number of speech fragments for each speaker, S, where $\varpi_{s}=(\frac{1}{n_{s}})\sum_{i=1}^{n_{s}}\omega_{i}^{s}$ is the average of each speaker’s I-vector. In order to preserve the inner product form of the cosine kernel, the feature mapping function, φ, was defined as Equation (9), as follows:

(9) $$\varphi (\omega )=B^{t}\omega$$

We applied the WCCN algorithm to the cosine kernel from the matrix B obtained by the Cholesky decomposition, $W^{-1}=BB^{t}$. The updated cosine kernel could then be calculated by Equation (10):

(10) $$k(\omega_{1},\omega_{2})=\frac{{\left(B^{t}\omega_{1}\right)}^{t}(B^{t}\omega_{2})}{\sqrt{{\left(B^{t}\omega_{1}\right)}^{t}(B^{t}\omega_{1})}\sqrt{{\left(B^{t}\omega_{2}\right)}^{t}(B^{t}\omega_{2})}}$$

In order to compensate for the variability between sessions, the WCCN algorithm normalized the cosine kernel function by using the intra-class covariance matrix, while ensuring the conservation of the spatial direction.

LDA has been widely used in the field of machine learning to analyze data and redefine new axes. It maximizes the difference between data and minimizes the difference within data categories, while achieving the goals of dimensionality reduction and classification. After modeling through I-vector, we treated each category as though they were composed recordings by the same speaker. We then defined the Rayleigh quotient (11) of LDA:

(11) $$J(v)=\frac{v^{t}s_{b}v}{v^{t}s_{\omega }v}$$

where $S_{b}$ is the difference between classes and $S_{\omega }$ is the difference within classes, assuming that in the spatial direction, v, we could find the maximized Rayleigh quotient by calculating the ratio between them, (12), (13), as follows:

(12) $$S_{b}=\sum_{s=1}^{S}(\omega_{s}-\varpi ){\left(\omega_{s}-\varpi \right)}^{t}$$

(13) $$S_{\omega }=\sum_{s=1}^{S}\frac{1}{n_{s}}\sum_{i=1}^{n_{s}}(\omega_{i}^{s}-\varpi_{s}){\left(\omega_{i}^{s}-\varpi_{s}\right)}^{t}$$

where S is the number of speakers, and the overall average vector of the speakers is equal to the empty vector. However, its average value was zero, in which $\varpi_{s}=(\frac{1}{n_{s}})\sum_{i=1}^{n_{s}}\omega_{i}^{s}$ was the average of the I-vector of each speaker, and $n_{s}$ is the number of speech fragments for each speaker, S. When we find the maximized Rayleigh quotient, we can define the projection matrix composed of the eigenvector with the largest eigenvalue. As shown in (14):

(14) $$S_{b}v=\lambda S_{\omega }v$$

where *A* is the projection matrix we obtained, and λ is the diagonal matrix of eigenvalues. We then introduced the I-vector into projection matrix *A* for calculation, and these two, $\omega_{1},\omega_{2}$, were I-vectors, and the new cosine kernel between them could be calculated by Equation (15):

(15) $$k(\omega_{1},\omega_{2})=\frac{{\left(A^{t}\omega_{1}\right)}^{t}(A^{t}\omega_{2})}{\sqrt{{\left(A^{t}\omega_{1}\right)}^{t}(A^{t}\omega_{1})}\sqrt{{\left(A^{t}\omega_{2}\right)}^{t}(A^{t}\omega_{2})}}$$

## 3. Image Encryption System

**Figure 3:**
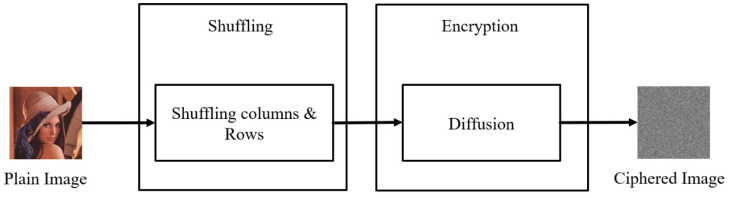
Two-stage encryption flowchart.

The classic image encryption system has a two-stage encryption. The first stage was rearrangement. The position of the pixels was rearranged (i.e., shuffled). The purpose of this was to eliminate the correlations between the pixels, making them harder to identify. The second stage was diffusion, which changed the pixel values, resulting in encryption. The pixel value distribution of the encrypted image was uniform, and the pixel value distributions of the encrypted image after this stage were very similar, making the image impossible to identify and improving the encryption security. For the two-stage encryption structure during the first stage, we input an image, converted the photo to grayscale (0~255), and then converted the pixels to a binary system and stored them as a matrix, M. To use the position matrix, M, we transferred the values to the position matrix, $M_{s}$, to rearrange them, and therefore the first stage of shuffling was complete. Next, we converted the two sets of data from the hyper-chaotic system (Lorenz system, Chen’s system) into a binary system, and XOR with $M_{s}$. The result of the unimodal mapping was subjected to a second XOR, and finally the biometric features were applied as the keys for the third XOR process. A two-level encryption procedure was carried out, and the two-stage encryption process (Figure 3) was also complete (diffusion).

### 3.1. Biometric Key and Initial Conditions

In this part, the user enters their account information, their password, and their input vocal signal. The system then converts the account password to the initial conditions through RFC 4648 Base32, as listed in Table 1; the input digits for the account information and password were limited to 10 and 16 bits, respectively. After the RFC 4648 Base32 conversion, *U_i_* referred to a 4-byte value that was derived by dividing the encoded user account into eight equal parts, with each part consisting of 2 bytes. The numerical equivalent of Base 32 was then used to represent this value. However, *P_i_* represented a 6-byte value obtained by dividing the encoded password into eight equal parts, with each part consisting of 3 bytes. The two most recent bytes in the last part of the password represented the remainders, which were denoted as R. Similar to *U_i_*, the numerical equivalent of Base 32 was used to represent both *P_i_* and *R*. The initial conditions were then used to drive the logistic map and hyper-chaotic system in order to iteratively generate values. The equation that produced the initial conditions could be expressed as follows:

(16) $${\mathrm{IC}}_{i}=\frac{U_{i}}{P_{i}\times R},\;i=1,\ldots ,8$$

RFC 4648 Base32 is an encoding mechanism that can convert data into symbols and numbers. This encoding system included base16, base32, base64, etc. Among them, base32 used 26 English letters, A–Z, numbers 2–7, and a total of 32 codes. RFC 4648 Base32 is considered the most widely used. The conversion table is shown in Table 1.

### 3.2. Logistic Map and Hyper-Chaotic System

**Figure 4:**
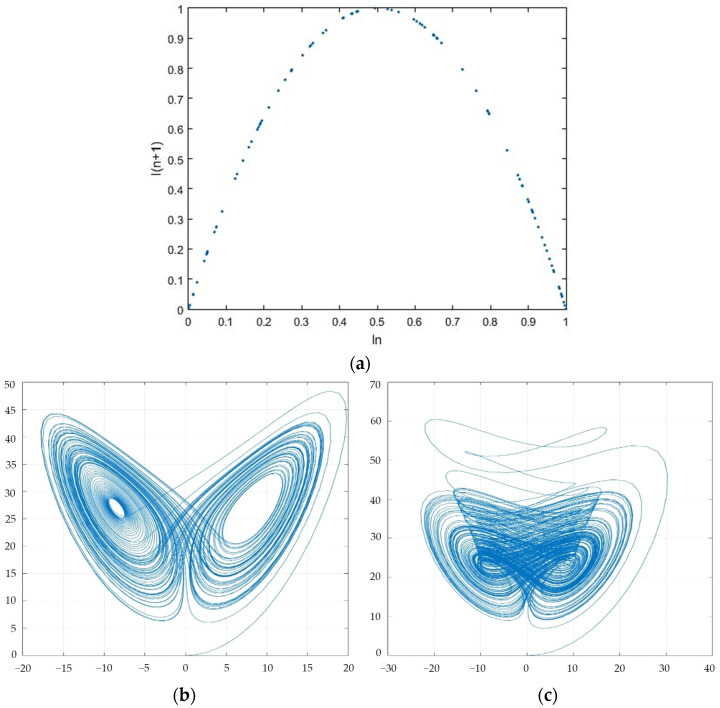
(**a**) Chaotic behavior of the logistic map; (**b**) Chaotic behavior of Lorenz system; (**c**) Chaotic behavior of Chen’s system.

The logistic map was an iterative function, which began with the initial value, $x_{i}$, of the variable and generated a series of values such as $x_{2}x_{3}$, …. Chaotic behavior could be generated by a very simple non-linear dynamic equation, such as that shown in Equation (17):

(17) $$x_{i+1}=rx_{i}(1-x_{i})$$

where i=0,1, … , ∞, $x_{i}$ is a number between 0 and 1 that will oscillate continuously, and the value exhibits non-linear behavior, making $x_{i}$ difficult to predict, and *r* = 4. Figure 4a shows its chaotic behavior.

Furthermore, in this study, we utilized the classical Lorenz system and Chen’s system as hyper-chaotic systems. These systems generate chaotic sequences with various combinations, which can then be utilized to introduce randomness into the encryption process. The classical Lorenz system can be described as follows:

(18) $$\{\begin{array}{l} {\dot{x}}_{1}=\sigma \left(x_{2}-x_{1}\right),\\ {\dot{x}}_{2}=-x_{1}x_{3}+\rho x_{1}-x_{2},\\ {\dot{x}}_{3}=x_{1}x_{2}-\beta x_{3}, \end{array}$$

where *x*_1_, *x*_2_, and *x*_3_ are system states; *σ*, *ρ*, and *β* are system parameters; and when *σ* = 10, *ρ* = 28, and *β* = 8/3, the system reveals chaotic behavior, as shown in Figure 4b. Furthermore, Chen’s system can be expressed as Equation (19):

(19) $$\{\begin{array}{l} {\dot{x}}_{4}=a\left(x_{5}-x_{4}\right),\\ {\dot{x}}_{5}=\left(c-a\right)x_{4}-x_{4}x_{6}+cx_{5},\\ {\dot{x}}_{6}=x_{4}x_{5}-bx_{6}, \end{array}$$

where *x*_4_, *x*_5_, and *x*_6_ are system states, *a*, *b*, and *c* are system parameters; and when *a* = 35, *b* = 3, and *c* = 28, chaos attractors are stimulated, which are provided in Figure 4c.

Hyper-chaotic systems are very sensitive to initial conditions. Even if the initial conditions are only slightly different, they will have a significant impact on the iteration results. Non-linear systems are also difficult to predict, but as long as they have the correct initial conditions, they can have the same iterative results and can, therefore, be used to generate the most encrypted data with high security.

### 3.3. Bit-Shuffling with Sliding Window

**Figure 5:**
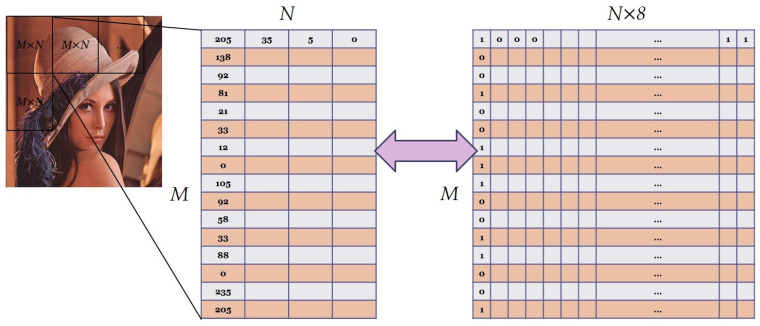
A schematic diagram for bit-shuffling with sliding window.

One of the significant properties of the images was the high correlation between adjacent pixels, which posed a challenge for image encryption. The shuffling stage of image encryption was responsible for eliminating these correlations by shuffling the pixel locations at the bit-level, in both horizontal and vertical directions. However, performing this operation on the entire image required substantial computational time and resources. To address this issue, we proposed a method of dividing the image into smaller sub-images using a sliding window size of *M × N* (in this study, we used a size of 16 × 4 pixels) and shuffling the content of each sub-image individually. This approach allowed for a more efficient generation of limited new locations for each bit number. The gray values of each pixel in the sliding window were transformed from decimal to binary, creating an *M × N ×* 8 bit-level sub-image. After the shuffling process, the sub-image was transformed back to the decimal values, and a new set of gray values for each pixel with low correlations to the adjacent pixels was obtained. This approach effectively eliminated the correlations between adjacent pixels while minimizing computational time and resources. A schematic diagram is provided in Figure 5.

To shuffle the pixel locations at the bit-level, we employed the logistic map in Equation (17) to generate new position matrices in the rows and columns. The system parameter for the logistic map was set at *r* = 4. By utilizing the logistic map, we were able to effectively generate the necessary new position matrices for the bit-level relocations, thereby enhancing the security of the encryption process. The shuffling process could be operated in the row direction and column direction separately, as discussed below:

(1). For column-shuffling, Equation (20) was applied via using the logistic map in Equation (17):
  (20) $$S_{column}=mod\left(floor\left(x_{n}\times 10^{14}\right),M\right)+1.$$
  where *x_n_* is the state of the logistic map and *S_column_* ϵ [1, *M*] is the new position for each binary number, which is a column vector with *M* × 1. This column vector, *S_column_*, could be generated for each column-shuffling process, until all the binary numbers had been rearranged. As a consequence, the total number of *S_column_* was *N* × 8.
(2). For row-shuffling, Equation (21) was applied using the logistic map in Equation (17), as follows:
  (21) $$S_{row}=mod\left(floor\left(x_{n}\times 10^{14}\right),N\times 8\right)+1.$$
  where *x_n_* is the state of the logistic map and *S_row_* ϵ [1, *N* × 8] is the new position for each binary number, which is a row vector with 1 × (*N* × 8*)*. This row vector, *S_row_*, could be generated for each row-shuffling process, until all the binary numbers had been rearranged. Moreover, the total number of *S_row_* was *M*.

### 3.4. Multi-Level Encryption Stage with Biometric Key and Selection Mechanism

**Figure 6:**
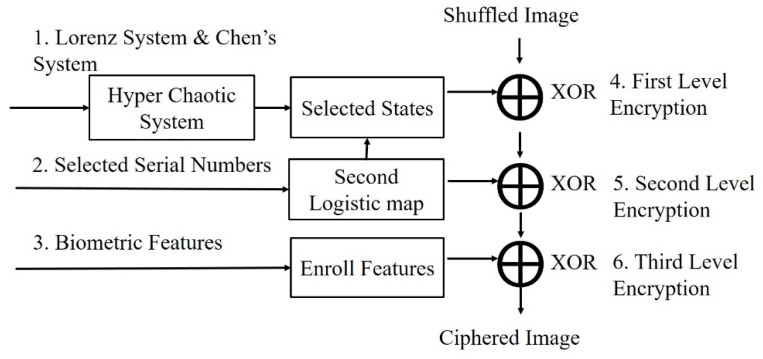
Three times XOR flowchart.

The shuffling images had been obtained during the preceding stages, and then the diffusion step, which constituted the encryption, could be executed. In this step, a three-level encryption process was performed, as shown in Figure 6.

The first-level process was obtained by iterating the initial conditions using the hyper-chaotic system in Equations (18) and (19), resulting in a total of 15 groups. A set of values was generated by using the logistic map in Equation (17), and one of the 15 groups was selected based on the generated values. The generated serial numbers (*SNs*) were within 0–14 and could be obtained by Equation (22), as follows:

(22) $$SN=mod\left(floor\left(x_{n}\times 10^{14}\right),14\right)$$

The different state combinations associated with the proposed 15 groups are provided in Table 2. The image was then processed using XOR, along with this set of results.

In accordance with the selected combinations in Equation (22), the first-level ciphering data generated via the two hyper-chaotic systems were obtained by Equation (23):

(23) $$P_{1i}=mod\left(floor\left(\left(\left|x_{i}\right|-floor\left(\left|x_{i}\right|\right)\right)\times 10^{14}\right),255\right)$$

where *i* = 1, 2, 3, 4 represent the four selected states, and |*x_i_*| returns the absolute value of *x_i_*. *Floor(x_i_)* returned the value of *x_i_* to the nearest integer less than or equal to *x_i_*, and *mod* returned the remainder after division. Finally, *P*_1*i*_ ϵ [0, 255] referred to the enciphering values applied during the first-level encryption process.

Furthermore, the values generated by the logistic map in the first-level process served as the key for the second encryption phase, which was the second-level encryption. Finally, for the third-level encryption, a user’s biometric features, or I-vectors, were employed for final image ciphering. The second- and the third-level encryption processes can be obtained via Equations (24) and (25), as follows:

(24) $$P_{2i}=mod\left(floor\left(x_{n}\times 10^{14}\right),255\right)$$

(25) $$P_{3i}=mod\left(floor\left(\left|\omega_{i}\right|\times 10^{14}\right),255\right)$$

The encryption process could be concluded via conducting an XOR operation on the image pixels shuffled by the permutation stage with the generated keys, which can be summarized as Equation (26), as follows:

(26) $$C_{i}=S_{i}⊕P_{1i}⊕P_{2i}⊕P_{3i}$$

where *i* = 1, 2, 3, 4, *P*_1*i*_, *P*_2*i*_, *P*_3*i*_ are the keys produced from the first-, second-, and third-level encryption processes, and *S_i_* is the image provided by the shuffling process. As shown in the selected machoism, as mentioned in Table 2, four pixels were ciphered in each round until all the content of the image had been protected. The process needed to be executed several times to encrypt the entire image. These iterations were performed via a selected 16 × 4 sliding window, and each initial condition was updated with the final results of each interaction.

### 3.5. Decryption Process

The decryption process in the proposed system closely mirrored the encryption algorithm, wherein the inverse operation was applied to the encrypted image to retrieve the plain image. It is worth noting that, in all stages of decryption, identical initial conditions and parameters had to be employed to achieve a successful decryption.

## 4. Experiment and Results Discussion

We used the voice database Pitch Tracking Database from the Graz University of Technology (PTDB-TUG) [26], which provided the microphone and laryngoscope signals of 20 native English speakers and the extracted pitch trajectory as a reference. The subjects read 2342 vocally rich sentences in the existing Massachusetts Institute of Technology Texas Instruments (TIMIT) corpus. We extracted the sound signals as the experimental database. The USC-SIPI image database is a collection of digitized images. It has primarily been used to assist in the research of image processing, image analysis, and machine vision. The first version of the USC-SIPI image database was released in 1977, and since then many new images have been added. We used four standard test charts, including Lena, F-16, Mandrill, and Peppers, as the smart images for image processing in this study.

### 4.1. Experiment Results of Speaker Verification System

**Figure 7:**
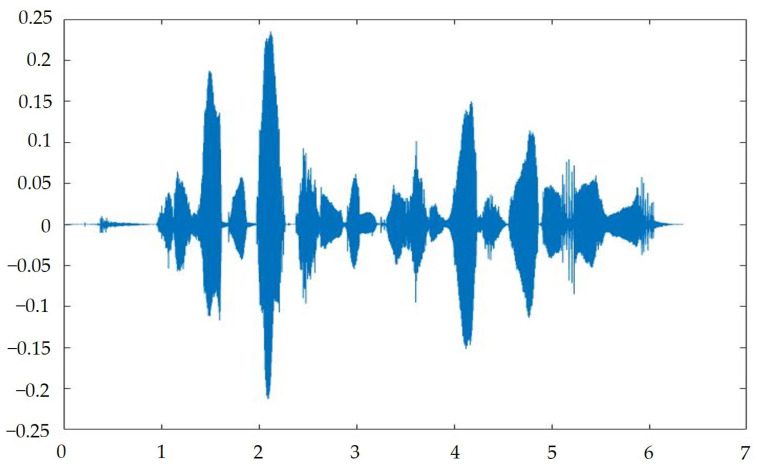
Input signal spectrum.

After creating a program in MATLAB, we input a set of sound signals, S, with a length of approximately 4 s, a sampling frequency of 16,000 Hz per second, a total of 63,488 data points, a sound frame length of 10 microseconds, and a sound frame number of 394. In the first step, we used MFCC to acquire the 15th order; the matrix dimension was 394 × 15. After the first Delta, the matrix increased by 394 × 15, and then the Delta matrix was increased by 394 × 15. After the logarithmic energy, 394 × 15 was added and the MFCC process had finished. We obtained 60 dimensional features, as shown in Figure 7.

**Figure 8:**
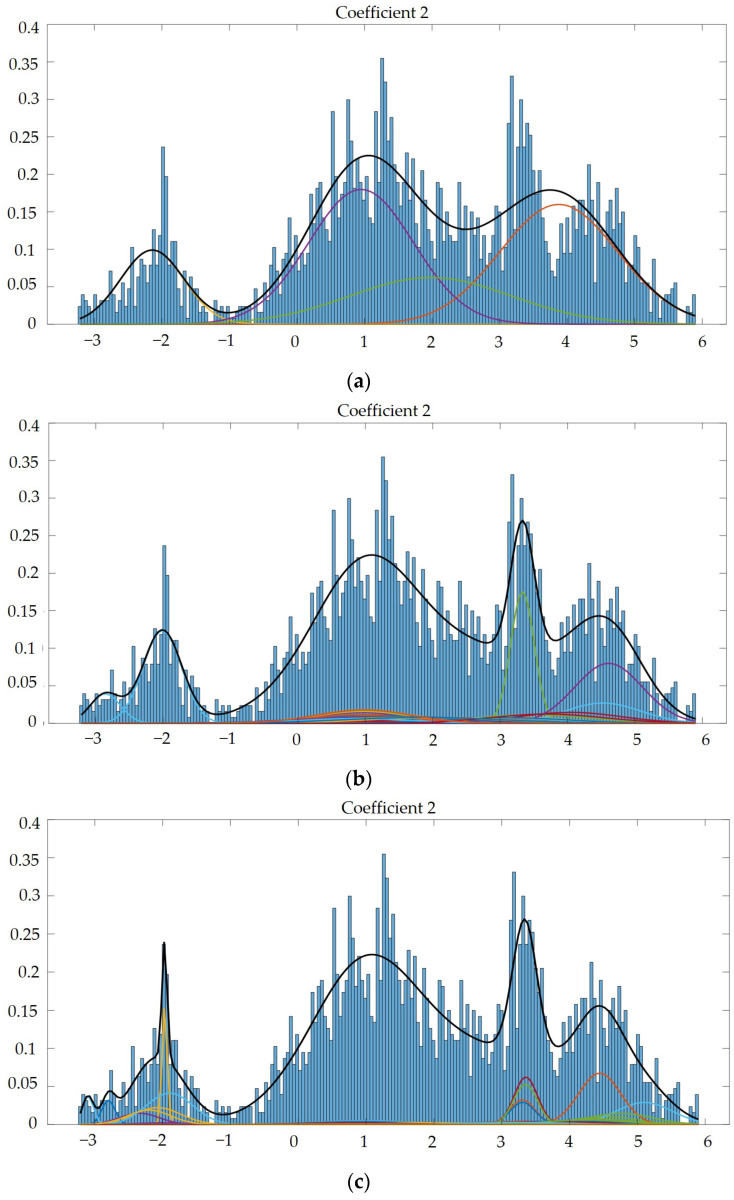

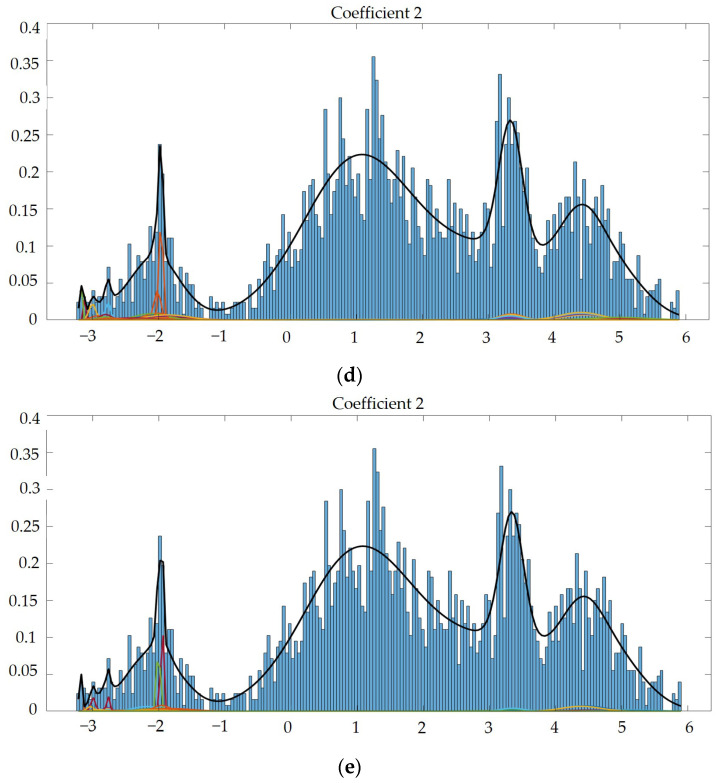
(**a**) Gaussian mixture model of coefficient 2 mixed with 4 Gaussian model components. (**b**) Gaussian mixture model of coefficient 2 mixed with 15 Gaussian model components. (**c**) Gaussian mixture model of coefficient 2 mixed with 512 Gaussian model components. (**d**) Gaussian mixture model of coefficient 2 mixed with 1024 Gaussian model components. (**e**) Gaussian mixture model of coefficient 2 mixed with 2048 Gaussian model components.

**Figure 9:**
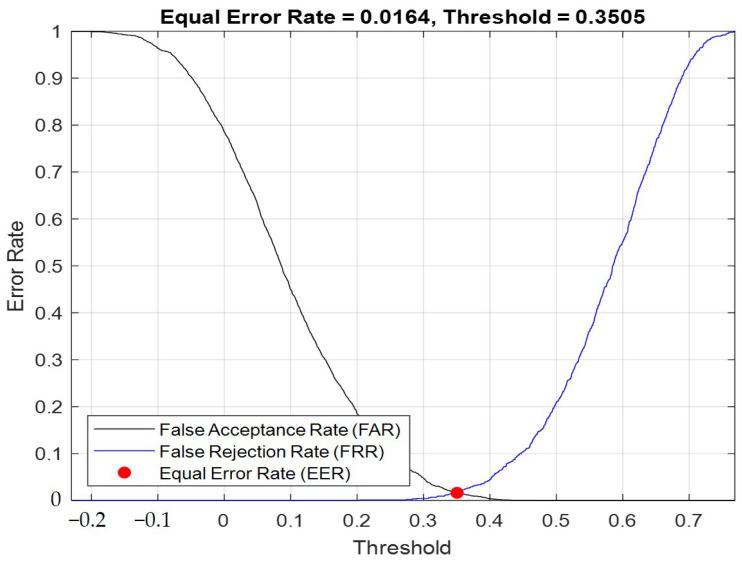
When Gaussian component = 2048, I-vector eigenvalue = 100. EER curve according to the threshold change.

Next, we used GMM to test the modeling function and extracted 2 dimensions from the 60-dimensional feature matrix completed by MFCC, in order to observe the modeling function. We used GMM to synthesize and model the parameters, so we could ascertain visually whether the established model was reasonable. The other lines were single Gaussian, and the thick black lines were the sum of multiple single Gaussians, as shown in Figure 8 and Figure 9. Through the different Gaussian components used to build acoustic models, we observed a phenomenon: the more Gaussian components, the more advantageous it was for building acoustic models, as the model was able to be more accurately fit to the data, as shown in [3]. The recommended number of Gaussian components was 2048, and the Gaussian component parameter of our next experiment was set to 2048.

Next, we analyzed the error rates of the speaker authentication system and observed the changes in the error rates by adjusting the discrimination criteria. After observing the changes, we could adjust our classifiers according to different application scenarios, such as authentication on mobile phones, which could be used frequently. If the classifier was too strict, it would result in ongoing access errors and reduced convenience. However, if it was applied in financial-related aspects, the classifier would need to be highly accurate in order to ensure that the registrant and the tester were the same person.

The false rejection rate indicated that the registered speaker and the test speaker were actually the same person, but the system mistakenly rejected the user’s authentication. The false acceptance rate referred to the scenario where the registered speaker and the test speaker were actually different people, but the system misjudged them as the same person. The equal error rate (EER) was determined when FRR and FAR were equal, and their intersection was the EER, which indicated that by adjusting the boundary, the FRR and FAR of the system could be changed according to the boundary to find the EER. The principle was that when the threshold was lowered (i.e., the degree of similarity between the registrant and the tester’s I-vector, cosine similarity), that is, less strict, the false acceptance rate increased. If the threshold was raised, which was equivalent to the authentication system becoming stricter, the false acceptance rate would decrease, but the false rejection rate would increase. Therefore, we hoped to identify the ideal compromise to determine the EER, where the false rejection rate would be equal to the false acceptance rate. We could adjust the EER to optimize the user experience. For example, in environments that require absolute security, we expected this system to be rigorous. However, in other scenarios when the security did not need to be as high, such as in situations involving frequent use, the authentication system could be less strict.

### 4.2. Experiment Results of Image Encryption System

**Figure 10:**
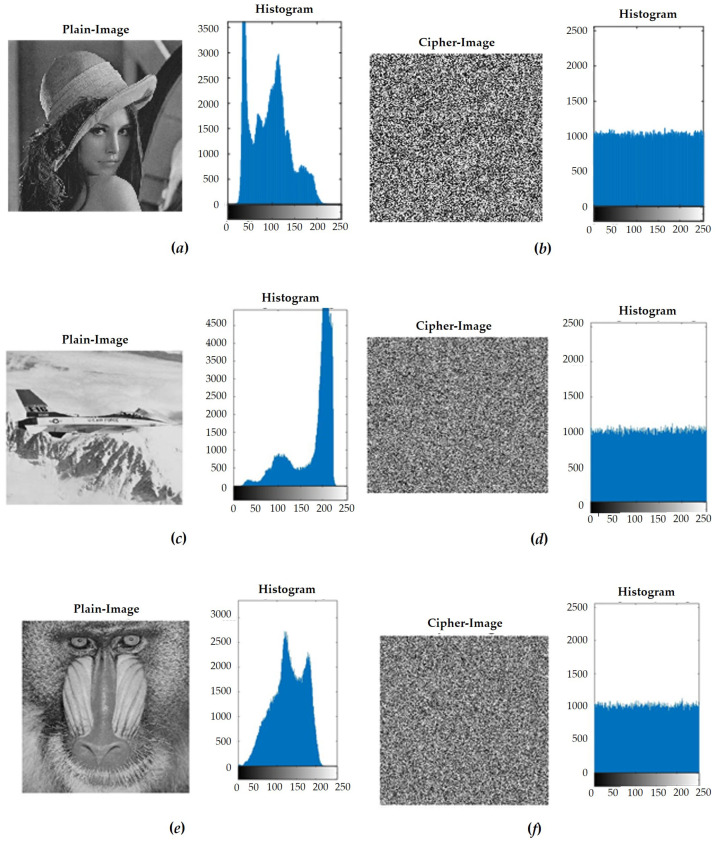

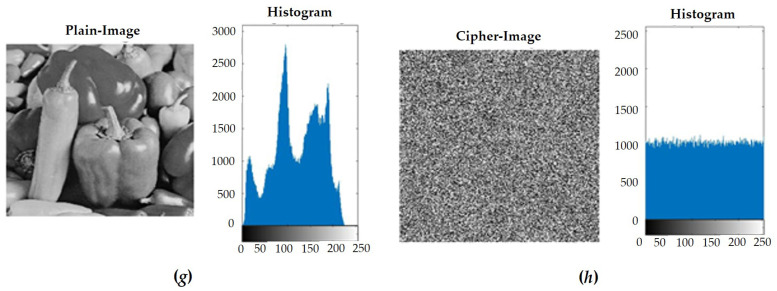
(**a**) Lena’s plain image and histogram of gray value. (**b**) Lena’s ciphered image and histogram of gray value. (**c**) F-16’s plain image and histogram of gray value. (**d**) F-16’s ciphered image and histogram of gray value. (**e**) Mandrill’s plain image and histogram of gray value. (**f**) Mandrill’s ciphered image and histogram of gray value. (**g**) Peppers’ plain image and histogram of gray value. (**h**) Peppers’ ciphered image and histogram of gray value.

In the classic two-stage encryption system, the first step was to shuffle. In the process of rearrangement, we transferred the image to the 8-bit binary system for rearrangement. Then, we set the gray value of the image back to 10 in the carry system, which could also be changed by observing the image with the naked eye and statistically analyzing its pixel composition. The second stage was diffusion. In this stage, we applied XOR to the image through the hyper-chaotic system, along with the value generated by the unimodal mapping, which directly changed the pixel values due to the pseudo-random system we used. Therefore, the values we had generated were uniformly randomly distributed, and the encrypted results also showed uniformly random results, which could also be obtained by observing the image with the naked eye and performing a statistical analysis of the gray values. Next, in order to ensure that the encryption system was sufficient, we calculated and encrypted photos with different structures and then observed and analyzed the results. We used the standard test chart of image processing. The source of the images used was a total of three sets of data as a comparison. The images were Lena, F-16, Mandrill, and Peppers. The simulation results are provided in Figure 10.

**Figure 11:**
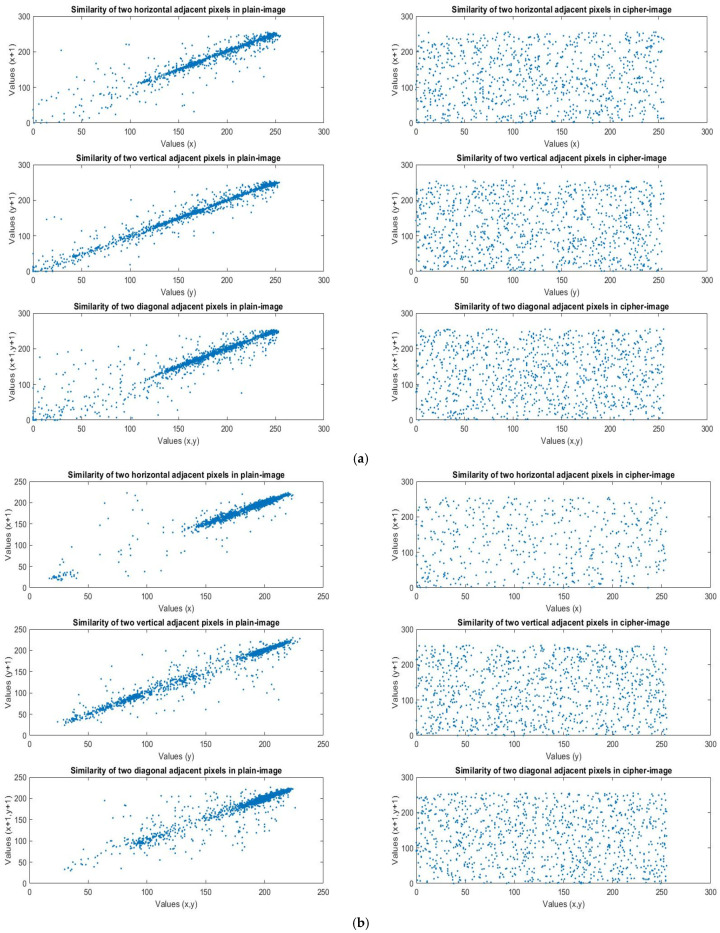

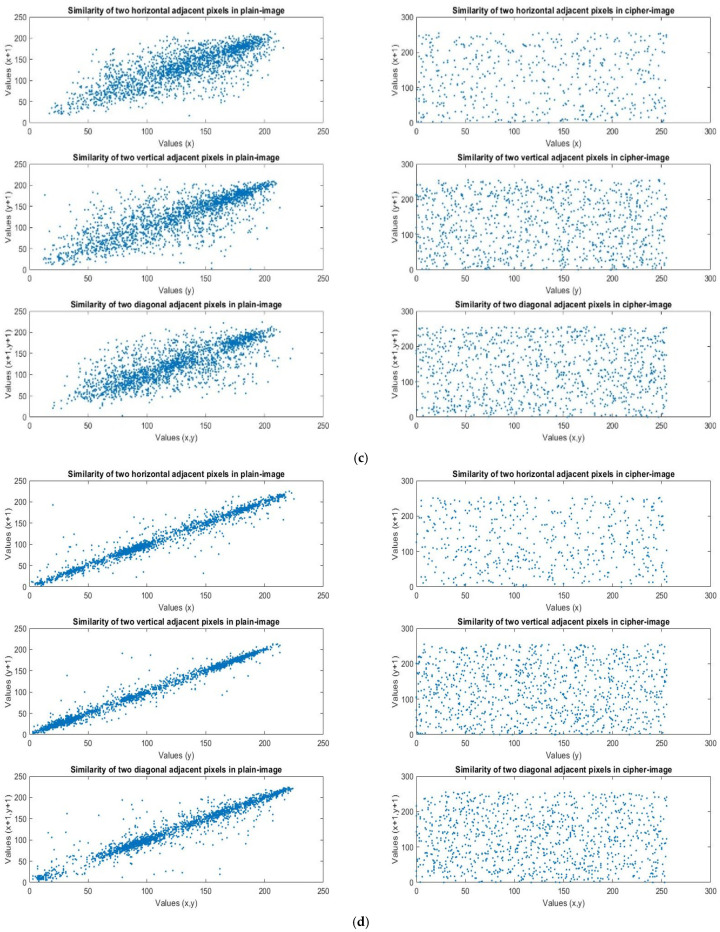
(**a**) Lena’s plain image and cipher image similarity in three directions. (**b**) F-16’s plain image and cipher image similarity in three directions. (**c**) Mandrill’s plain image and cipher image similarity in three directions. (**d**) Peppers’ plain image and cipher image similarity in three directions.

We analyzed the correlational coefficients based on the encrypted results of the four images with different structures, as shown in Figure 11. The purpose of the shuffle was to eliminate the relationships between adjacent pixels, so that the information of the plain image would remain hidden. We analyzed the correlations between two adjacent pixels in three directions. When the correlation was lower, the relationship between the pixels was weaker, and the effect of the rearrangement and encryption was better. The three directions were vertical, horizontal, and diagonal. Table 3 is based on the correlational coefficient analysis of the four images. We observed that the pixels, before encryption, had strong associations with each other, but after encryption, the relationship between the pixels was destroyed and the correlational coefficient was very low. No information could be obtained from the encrypted image, so the encryption effect was very good, as shown in Table 3.

In addition, we conducted a comparative analysis of our proposed method with other algorithms that had been previously proposed in the literature, using the Lena image as a reference. The results of this comparison are presented in Table 4, which shows that our proposed system was able to completely eliminate the correlations between adjacent pixels. Furthermore, we extended our analysis to include larger images in order to explore the feasibility of our approach on images of varying sizes. The results indicated that our method was successful in achieving the main objective of eliminating the correlations between pixels, with values approaching zero for both small and large images.

## 5. Discussion and Limitations

In this article, a framework for authentication and personal image protection aimed to connect personal information with data security and utilized voice samples to verify the user. Personal information, such as user account information, password, and voice, were applied to further generate the related key for image encryption, according to a classical two-stage encryption process. The simulation results showed that the proposed image encryption process was effective for ciphering the contents of an image, and the high correlations between different adjacent pixel pairs were also destroyed. 

For more information, the current version was an independent version that could only be realized for personal usage. For example, a user could register the proposed system for their user account information, password, and unique voice print, and the image they upload into a system could then be protected. However, the current version did not have integration mechanisms for users to transmit their uploaded images to other users securely, and the encryption and decryption processes for transmission and receiving need to be redesigned. This would require more complicated processes being developed. For example, a safe channel should be constructed, and the biometric keys from the two different users would have to be combined and employed simultaneously. In addition, the possibilities of the practical application of this framework for interoperability and messaging patterns [40] should be considered and explored in the future.

## 6. Conclusions and Future Directions

In this article, we presented a framework for authentication and personal image protection that aimed to connect personal information with data security. The main contributions of this work were the following:

(1) Development of a framework for the authorization and protection of personal images: We presented a comprehensive framework that combined user account information, passwords, and personal I-vectors as keys for encrypting image content. We established a connection between personal information and data security.
(2) Integration of speaker verification system and image encryption system: The framework incorporated two main systems. The speaker verification system prompted the user to provide a short voice sample for speaker identification. The personal voice features, along with user account information and passwords, were then transferred to the image encryption system. This integration enhanced the security and robustness of the encryption process.
(3) Utilization of biometrics and hyper-chaotic systems for image encryption: The user’s account information and passwords were utilized to generate the initial conditions for hyper-chaotic systems, which produced private keys for image-shuffling and ciphering. Furthermore, biometric features, such as voice biometrics, were employed to enhance the robustness of the encryption process.
(4) Demonstrated effectiveness and efficiency: The proposed smart image encryption algorithm was evaluated using standard test images, and the results showed acceptable lower correlations in the ciphered images. The framework demonstrated its effectiveness for protecting image content while maintaining efficiency in the encryption process.

The proposed method for using a speaker-verification system to drive image encryption and decryption may have potential applications in different fields where the security and privacy of personal data are crucial. For example, this method could be used to protect classified information and prevent unauthorized access to sensitive data. In financial institutions, it could be used to secure customer information and transactions. In healthcare, it could be used to protect patient data and maintain confidentiality. Additionally, this method could be implemented on personal devices, such as smartphones and laptops, to secure personal information and prevent theft or misuse. The proposed method offers a new layer of security that relies on the uniqueness of a user’s voiceprint, making it difficult for unauthorized users to access encrypted data, and we will continue to improve the proposed system in future work.

## Figures and Tables

**Table 1:** RFC 4648 base32 conversion table.

Value	Symbol	Value	Symbol	Value	Symbol
0	A	11	L	22	W
1	B	12	M	23	X
2	C	13	N	24	Y
3	D	14	O	25	Z
4	E	15	P	26	02
5	F	16	Q	27	03
6	G	17	R	28	04
7	H	18	S	29	05
8	I	19	T	30	06
9	J	20	U	31	07
10	K	21	V		

**Table 2:** Different state combinations of the proposed 15 groups.

Serial Number	Combination of States	Serial Number	Combination of States
0	(*x*_1_, *x*_2_, *x*_3_, *x*_4_)	8	(*x*_1_, *x*_3_, *x*_5_, *x*_6_)
1	(*x*_1_, *x*_2_, *x*_3_, *x*_5_)	9	(*x*_1_, *x*_4_, *x*_5_, *x*_6_)
2	(*x*_1_, *x*_2_, *x*_3_, *x*_6_)	10	(*x*_2_, *x*_3_, *x*_4_, *x*_5_)
3	(*x*_1_, *x*_2_, *x*_4_, *x*_5_)	11	(*x*_2_, *x*_3_, *x*_4_, *x*_6_)
4	(*x*_1_, *x*_2_, *x*_4_, *x*_6_)	12	(*x*_2_, *x*_3_, *x*_5_, *x*_6_)
5	(*x*_1_, *x*_2_, *x*_3_, *x*_4_)	13	(*x*_2_, *x*_4_, *x*_5_, *x*_6_)
6	(*x*_1_, *x*_3_, *x*_4_, *x*_5_)	14	(*x*_3_, *x*_4_, *x*_5_, *x*_6_)
7	(*x*_2_, *x*_3_, *x*_4_, *x*_6_)		

**Table 3:** Four standard test images, Lena, F-16, Mandrill, and Peppers, plain images and ciphered images after a correlational coefficient analysis.

		Correlational Coefficient		
Name	Image Type	Horizontal	Vertical	Diagonal
Lena	Plain image	0.967504	0.973323	0.958654
	Ciphered image	0.050908	−0.019047	0.039765
F16	Plain image	0.962689	0.975145	0.934036
	Ciphered image	0.035941	0.009310	−0.021671
Mandrill	Plain image	0.819316	0.776839	0.728311
	Ciphered image	0.000468	−0.020855	0.012727
Peppers	Plain image	0.977299	0.984698	0.960850
	Ciphered image	−0.011531	0.021001	0.017306

**Table 4:** Comparison results of the correlational coefficient test (Lena).

		Correlational Coefficient		
Name	Image Type	Horizontal	Vertical	Diagonal
Our Method	Plain image	0.967504	0.973323	0.958654
	Ciphered image	0.050908	−0.019047	0.039765
Ref. [37]	Plain image	0.967504	0.973323	0.958654
	Ciphered image	−0.079639	0.016615	0.003277
Ref. [38]	Plain image	0.967504	0.973323	0.958654
	Ciphered image	0.001400	0.017100	0.005400
Ref. [39]	Plain image	0.967504	0.973323	0.958654
	Ciphered image	0.001906	0.003817	−0.001948

## Data Availability

No new data were created in this paper.
